# Measures and models for causal inference in cross-sectional studies: arguments for the appropriateness of the prevalence odds ratio and related logistic regression

**DOI:** 10.1186/1471-2288-10-66

**Published:** 2010-07-15

**Authors:** Michael E Reichenheim, Evandro SF Coutinho

**Affiliations:** 1 Department of Epidemiology, Institute of Social Medicine (IMS), Rio de Janeiro State University (UERJ), Rio de Janeiro, Brazil; 2 Department of Epidemiology and Quantitative Methods, National School of Public Health Sergio Arouca (ENSP), Oswaldo Cruz Foundation (Fiocruz), Rio de Janeiro, Brazil

## Abstract

**Background:**

Several papers have discussed which effect measures are appropriate to capture the contrast between exposure groups in cross-sectional studies, and which related multivariate models are suitable. Although some have favored the Prevalence Ratio over the Prevalence Odds Ratio -- thus suggesting the use of log-binomial or robust Poisson instead of the logistic regression models -- this debate is still far from settled and requires close scrutiny.

**Discussion:**

In order to evaluate how accurately true causal parameters such as Incidence Density Ratio (IDR) or the Cumulative Incidence Ratio (CIR) are effectively estimated, this paper presents a series of scenarios in which a researcher happens to find a preset ratio of prevalences in a given cross-sectional study. Results show that, provided essential and non-waivable conditions for causal inference are met, the CIR is most often inestimable whether through the Prevalence Ratio or the Prevalence Odds Ratio, and that the latter is the measure that consistently yields an appropriate measure of the Incidence Density Ratio.

**Summary:**

Multivariate regression models should be avoided when assumptions for causal inference from cross-sectional data do not hold. Nevertheless, if these assumptions are met, it is the logistic regression model that is best suited for this task as it provides a suitable estimate of the Incidence Density Ratio.

## Background

Mainstream books devoted to organizing knowledge on epidemiological methods used to emphasize the study of the distribution of health events according to person, time and place [1,2]. Following a period when vital statistics were the main data sources for this aim, cross-sectional studies began to play an important role in this field providing prevalence aggregate and group-specific estimates.

Acknowledging the limitations of prevalence *vis-à-vis *incidence estimations in some circumstances, from the 1980's, a particular literature focused on cross-sectional data as a means to indirectly obtain the estimations of incidence rates [3-8]. Simultaneously, following a growing interest of the epidemiological community on causal inference [9,10], cross-sectional studies were not only accepted as a way of estimating prevalences, but given certain conditions, also as a suitable design for investigating causal relationships. Since the 1990's, papers recognizing the analytical role of epidemiological surveys have been concerned with two issues: (i) which measure would be appropriate for capturing the contrast between exposure groups, whether the prevalence ratio (PR) or the prevalence odds ratio (POR); and (ii) which model should be used for estimating these quantities in the multivariate context.

The first issue raised a debate which split two camps. Whereas Strömberg [11,12] favored the POR, Lee & Chia [13,14], Lee [15], Axelson et al. [16,17] and Thompson et al. [18] argued that the estimator was difficult to interpret and communicate; was very discrepant from PR when outcomes were common; and that the conditions when POR estimates the incidence density ratio (IDR) -- stationarity and equal duration of disease -- were hardly met. Reservations were also expressed on the account that the POR was a numerical mimic to other effect measures; and was misinterpreted as cumulative incidence ratio (CIR) in the presence of common outcomes.

Although Pearce [19] -- again favoring the POR -- revisited the dispute only a few years ago, the debate *per se *seemed to have died away, being outshined by the second contention, namely, as to which methods would be best to model prevalence data. Since surveys most often involve frequent events, several authors called up the 'rare disease assumption' [20,21] and argued that the POR obtained by logistic regression model would thus overestimate the PR. As an extension, statistical models other than logistic regression have been proposed to estimate PR, namely, the log-binomial, Poisson or Cox models with robust variance estimates [18,22-26]. Several studies have taken this on and have been effectively using these models to handle data arising from cross-sectional data and envisaging control for confounding variables. For instance, a preliminary and tentative literature search in Medline (October 8, 2009) using the keywords ("prevalence ratio*"[All Fields] OR "cross-sectional"[All Fields]) AND (Poisson[All Fields] OR "log-binomial"[All Fields]) AND "humans"[MeSH Terms] found 444 references of this kind. Conspicuously, the numbers increased from 20 papers between 1990-1994, to 262 in the 2005-2009 period.

It is our contention that this perspective is essentially misguided and that a discussion on the best model only makes sense if preceded by a thorough debate about what is actually sought with a cross-sectional study: estimating the magnitude of a condition in a population or making causal inference? The purpose of this paper is thus to revisit a dispute that, though hardly new by any means, is far from settled. In fact, our motivation has been this growing literature that utilizes multivariate models for PR to address relationships between a 'dependent' and several 'independent' variables, yet in our view without a clear-cut supporting rationale behind it.

## Discussion

To address our contention, this section is organized as follows. The first subsection provides a brief review regarding the purposes of cross-sectional studies, with a particular eye on identifying the specific situations when measures of association based on information gathered from cross-sectional studies are in fact wanted and/or required, and when multivariate modeling procedures are hence due. The ensuing subsection dwells on the relation between prevalence and incidence ratio measures. It starts by presenting the necessary conditions whereby a cross-sectional study may be able to provide measures of effect representing causal parameters. Next, in preparation to the proposed scenarios and discussions that follow, an outline on the formal relations between measures originating from cross-sectional approaches (PR and POR) and respective relations to causal parameters -- the cumulative incidence ratio and the incidence density ratio (IDR) -- are provided. In sequence we create several scenarios that pragmatically assume that data come from surveys (as in so many real instances) and enquire whether and how the actual obtained measures (PR or POR) effectively inform about either CIR or IDR. The next subsection offers the rationale for choosing a suitable multivariable model, followed by a subsection building upon the arguments provided previously and proposing a decision tree for analyzing cross-sectional data. Finally, in the light of our own insights, the last subsection of the Discussion revisits some of the points mentioned in the Background section for and against the measures and models used for causal inference in cross-sectional studies.

### Purposes of a cross-sectional studies

Before delving into a debate about which multivariate model to use, a key issue in deciding between contrast measures (PR or POR) concerns the underlying reason for actually wanting to obtain ratios involving estimates in cross-sectional studies. To answer this question one needs to summon up the two main purposes for carrying out health surveys.

The most usual and uncontroversial is to provide overall and group-specific prevalences of a particular health event in a given population, usually with an outlook to help organizing resources and to guide decision-making processes. In this particular case, estimating a PR -- or more precisely, a single-value estimate contrasting the prevalences obtained in two population strata -- may not be of much interest. Concretely, what would a health officer make of being informed that the PR equals 2.0 in a particular population? This value could signify, for example, that the magnitude of an event of interest in two different strata is 20% and 10%, but may also relate to prevalences of 0.02% and 0.01%, respectively. Clearly, these are very different scenarios in terms of relevance and resulting health actions, which an omnibus measure as this PR of 2.0 simply fails to portray.

This also reminds us that when causal inference is not desired or possible (*c.f*. subsection "Structuring conditions" further on) the knowledge regarding tangible magnitudes of specific prevalences is much more informative than their ratio. It follows that statistical modeling to 'adjust for several variables' may also provide little help here and is unwarranted in such circumstances. An exception is when it is unfeasible to directly calculate estimates of prevalence due to data sparseness within subgroups, especially if there are specific target groups to be identified for further action. In order to get around this problem, it is possible to resort to multivariate modeling to predict or project prevalences (probabilities) using, for instance, a logistic model and thereafter applying the anti-logit function $Pr\left(P\right)={\left(1+\exp-(\beta_{0}+\sum_{i=1}^{k}\beta_{i}X_{i})\right)}^{-1}$, with *k *variables describing patterns of characteristics in these subgroups. Still, it important to realize that in this particular situation one is neither modeling nor interested in any effect (ratio) measure, but rather in the actual probabilities/prevalences occurring in -- or rather, projected for -- certain subgroups of interest.

Another purpose of surveys is to uncover causal relationships. Although longitudinal study designs are better suited for this aim, cross-sectional studies have often been used to answer causal questions, mostly for pragmatic reasons such as unavailability of incidence data, reducing cost and duration of a study, and sometimes because of ethical constraints, as for instance, when a detected exposure unequivocally needs immediate intervention, rendering a 'neutral' follow up unsustainable.

Now, if the purpose of a survey is to address a causal relation, one of the key issues required for dealing with observational data concerns the modeling procedure. Yet, ahead of engaging in modeling the 'natural estimator' yielded in a survey (e.g., PR for some), one has to step back and ask what is really being achieved by controlling for several co-variables. Specifically in the context depicted here, the question is why a researcher would want to obtain the average prevalence ratio accounting for the other variables in the model. One answer would be to 'recover' a counterfactual estimation that contrasts the exposed with 'themselves if unexposed' regarding the outcome of interest, which is only empirically achieved by comparing the exposed with the actual unexposed, once the effect of other relevant factors are explained away. This is evidently (and not surprisingly to most readers) a way to deal with confounding within the perspective of what has been labeled the potential-outcome model [27,28]. If this is indeed a reasonable model, what would the required estimator(s) then be? Would a ratio of two prevalences (which, *inter alia*, may conflate incidence and duration of the event) suffice or would its ultimate aim be to estimate risk ratios (CIR) or rate ratios (IDR) given both are recognizably 'true' causal parameters and largely recommended as the appropriate quantities to be attained [9,29]? If the latter, an essential task is to scrutinize as to when and how a survey is effectively able to produce measures that are capable of representing any of these two causal parameters.

### Relating prevalence and incidence ratio measures

#### Structuring conditions

At the outset, five conditions are necessary for a cross-sectional approach to be able to investigate an etiological hypothesis, and without which any attempt to relate an ensuing estimator to either the CIR or IDR breaks down. For one, the population must be in steady state over the study period (stationary). In this case, within any given period of time, the size of the population needs to be constant across the exposure groups, as well as in regards to any other co-variable used in the modeling process. Secondly, no selective survival is allowable, i.e., the probability of withdrawal or death from the outcome under study or from other related causes may not be different across exposure groups. Thirdly, the mean duration of the outcome must be the same regardless of exposure group, that is, the exposure may not differentially influence the survival or recovery probabilities. Fourthly, no reverse causality is allowed, i.e., the outcome being modeled may not reciprocally cause (influence) the exposure status in any way. Lastly, the temporal directionality from the exposure to the outcome must be sustainable, either theoretically (e.g., if a lifelong attribute is studied as the exposure for a recent outcome event) or by means of a thorough data collection procedure that assures the exposure as an antecedent of the outcome (e.g., in a study on the effects on child birth, recalling at birth a past exposure during pregnancy) [9,30].

Once these criteria are exhaustively met, the next step consists of inspecting the conditions whereby the estimates obtained in cross-sectional studies capture causal parameters or, in contrast and most importantly, under which circumstances they fall apart.

#### Formal relations between measures

Recalling Kleinbaum et al. [9] and notation therein, let $R_{(t_{0},t),i}=CI_{(t_{0},t),i}$ be the risk or the cumulative incidence of an outcome of interest (e.g., disease, illness) occurring in stratum *i *within a time interval Δ*t *= (*t*_0_, *t*); *ID*_*i *_be the respective incidence density; ${\bar{T}}_{i}$ be the mean duration of the outcome; and *P*_*i *_the point prevalence measured (obtained) through a cross-sectional approach.

Dropping the subscript (*t*_0_, *t*) for ease of notation, define

(1) $$CI_{i}=1-\exp\left[-(ID_{i}\;\Delta t)\right]$$

and thus conversely

(2) $$ID_{i}=\frac{\ln\left[1/\left(1-CI_{i}\right)\right]}{\Delta t}.$$

Since, generically, the relation between incidence density and prevalence is

(3) $$P_{i}=\frac{ID_{i}{\bar{T}}_{i}}{ID_{i}{\bar{T}}_{i}+1},$$

the ensuing prevalence ratio is

(4) $$PR=\frac{P_{1}}{P_{0}}=\frac{ID_{1}{\bar{T}}_{1}/\left(ID_{1}{\bar{T}}_{1}+1\right)}{ID_{0}{\bar{T}}_{0}/\left(ID_{0}{\bar{T}}_{0}+1\right)}.$$

Hence, if according to equation (2) one has *ID*_0 _= [ln(1/(1 - *CI*_0_))]/Δ*t *for the unexposed and *ID*_1 _= [ln(1/(1 - *CI*_1_))]/Δ*t *for the exposed, further substituting these quantities into equation (3), the ensuing prevalences are, respectively,

(5) $$P_{0}=\frac{\left(\ln\left[1/(1-CI_{0})\right]/\Delta t\right)\;{\bar{T}}_{0}}{\left(\left(\ln\left[1/(1-CI_{0})\right]/\Delta t\right)\;{\bar{T}}_{0}\right)+1}$$

(6) $$P_{1}=\frac{\left(\ln\left[1/(1-CI_{1})\right]/\Delta t\right)\;{\bar{T}}_{1}}{\left(\left(\ln\left[1/(1-CI_{1})\right]/\Delta t\right)\;{\bar{T}}_{1}\right)+1}$$

and, thus, the prevalence ratio (PR) may also be written as a function of the underlying risks (*CI*_*i*_) and outcome durations ${\bar{T}}_{i}$ as

(7) $$PR\text{ = }\frac{\ln\left[1/(1-CI_{1})\right]\cdot {\bar{T}}_{1}\cdot \left(\ln\left[1/(1-CI_{0})\right]\cdot {\bar{T}}_{0}+\Delta t\right)}{\ln\left[1/(1-CI_{0})\right]\cdot T_{0}\cdot \left(\ln\left[1/(1-CI_{1})\right]\cdot {\bar{T}}_{1}+\Delta t\right)}$$

From equation (3), *ID*_*i *_may be expressed as a function of an estimated prevalence

(8) $$ID_{i}=\frac{P_{i}}{(1-P_{i}\text{) }{\bar{T}}_{i}}$$

and, therefore,

(9) $$IDR=\frac{ID_{1}}{ID_{0}}=\left(\frac{P_{1}/\left(1-P_{1}\right)}{P_{0}/\left(1-P_{0}\right)}\right)\frac{{\bar{T}}_{0}}{{\bar{T}}_{1}}$$

Note that the *IDR *given in equation (9) may also be written as a function of the prevalence odds-ratio

(10) $$IDR=POR\left(\frac{{\bar{T}}_{0}}{{\bar{T}}_{1}}\right)$$

and when the outcome durations are the same in both strata (exposed and non-exposed), the *IDR *equals the *POR*.

The *CI_i _*may also be expressed from the prevalence *P*_*i*_. Solving equation (5) or (6), generically for stratum *i*, one has

(11) $$\;CI_{i}=1-\exp\left(-\frac{P_{i}\Delta t}{T_{i}-P_{i}T_{i}}\right)$$

and therefore,

(12) $$CIR=\frac{1-\exp\left(-\frac{P_{1}\Delta t}{T_{1}-P_{1}T_{1}}\right)}{1-\exp\left(-\frac{P_{0}\Delta t}{T_{0}-P_{0}T_{0}}\right)}$$

All results in the following subsection (including figures) were obtained through an *ad hoc *Stata^® ^program (epiconv ado-file) based on the above relations. The routine may be obtained with one of the authors (MER) on request.

#### Exploring several scenarios

The scenarios that follow assume that data are collected through cross-sectional approaches and that the ratios of effectively measured prevalences between exposed and unexposed are always 2.0. Bearing a causal outlook, the fundamental issue concerns the interpretation in terms of the experience of the population under investigation. In the light of some or even all of the conditions presented (*c.f*. subsection "Structuring conditions"), how are the estimates to be read? What do they signify in terms of CI and ID, and by extension, their related effect measures CIR and IDR?

The scenarios portrayed in Table 1 vary according to whether the outcomes of interested are *(i) *rare or frequent events; *(ii) *whether their average durations are long or short; and *(iii) *if they are equal or unequal according to exposure group. The time units and prevalences specified in the scenarios are described in the table footnotes and are jointly meaningful. Note that given the specified values -- *P_1 _*and *P_0_*; *T_1 _*and *T_0_*; and *Δt --*, the IDR and CIR are specifically obtained through equations (9) and (12), respectively.

**Table 1:** Eight scenarios depicting cross-sectional studies carried out in different underlying conditions, yet all uncovering prevalence ratios between exposed and non-exposed groups of 2.0.

Scenario	Type of outcome event	Duration of outcome (${\bar{T}}_{i}$)	IDR *		CIR **	
1	Rare	Long and equal	2.053	(+2.6)	2.025	(+1.2)
2	Rare	Long and unequal	0.205	(-89.7)	0.227	(-88.6)
3	Rare	Short and equal	2.053	(+2.6)	1.809	(-9.5)
4	Rare	Short and unequal	0.205	(-89.7)	0.443	(-77.8)

5	Common	Long and equal	3.000	(+50.0)	2.230	(+11.5)
6	Common	Long and unequal	0.300	(-85.0)	0.656	(-67.2)
7	Common	Short and equal	3.000	(+50.0)	1.037	(-48.1)
8	Common	Short and unequal	0.300	(-85.0)	1.000	(-50.0)

* In brackets: % bias (IDR) calculated as [-(*PR *- *IDR *)/*PR*]*100, where PR = 2.0 is fixed.

** In brackets: % bias (CIR) calculated as [-(*PR *- *CIR *)/*PR*]*100, where PR = 2.0 is fixed.

Time units used in the scenarios

Average duration of outcome long and equal → *T_1 _*= *T_0 _*= 1

Average duration of outcome long and unequal → *T_1 _*= 1; *T_0 _*= 0.1

Average duration of outcome short and equal → *T_1 _*= *T_0 _*= 0.1

Average duration of outcome short and unequal → *T_1 _*= 0.1; *T_0 _*= 0.01

Time interval for projecting CIR → *Δt *= 1

Prevalences according to exposure group and outcome frequency

Rare outcome → *P_1 _*= 0.05; *P_0 _*= 0.025

Common outcome → *P_1 _*= 0.5; *P_0 _*= 0.25

Overall, the PR estimate is only consistent with the IDR and the CIR in very restricted situations. Table 1 shows that there is only proximity when the outcome is rare and its duration long and alike across exposure strata (scenario 1). In a still more constrained condition -- when the outcome is rare, short and of equal duration (scenario 3) -- only the IDR is numerically compatible with the PR, whereas the CIR is already quite far-off (9.5% attenuation).

**Figure 1:**
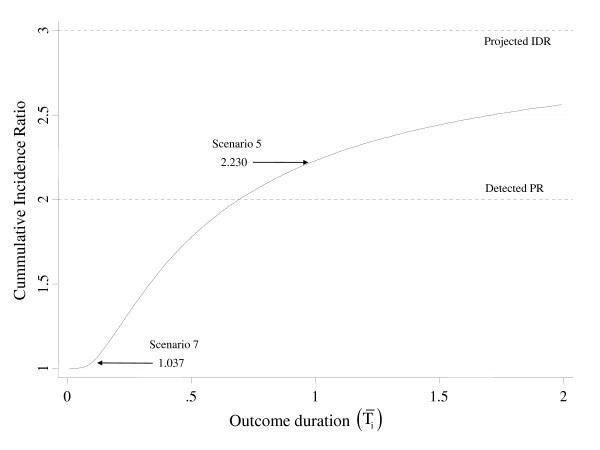
**Projected Cumulative Incidence Ratio (CIR) according to increasing disease durations (${\bar{T}}_{i}$), given surveys uncovering P_1 _= 0.5 and P_0 _= 0.25 (PR = 2.0), and Δt = 1**.

The shortcoming of the PR *vis-à-vis *CIR is plainly depicted in Figure 1, which shows how the latter varies according to the duration of outcome, given fixed prevalences among exposure groups and time period (Δt) concerning the risks involved in the projected CIR. Bearing that the straight dotted line in the centre of the figure indicates the 'constant' PR = 2.0 that would be uncovered in several cross-sectional studies, note that the CIR is met in just a very narrow range of outcome durations, strictly reaching equality only when ${\bar{T}}_{1}={\bar{T}}_{0}=0.69$. Note that arrows are indicative of the combinations shown in scenarios 5 and 7 of Table 1.

Although less common, one could contend that rather than standing for the CIR, the PR may capture the IDR instead. The dotted line at the top of Figure 1 shows that the projected IDRs are quite afar from the PR. In this scenario the latter blatantly underestimates the former by 50% throughout the ${\bar{T}}_{i}$ range, clearly not supporting the above proposition. Although one may argue that the PR tends to converge to the IDR as the outcome event gets rarer, the question remains as to 'how rare should an outcome be' in order to enable one to accept the PR as a consistent proxy to the causal effect parameter.

**Figure 2:**
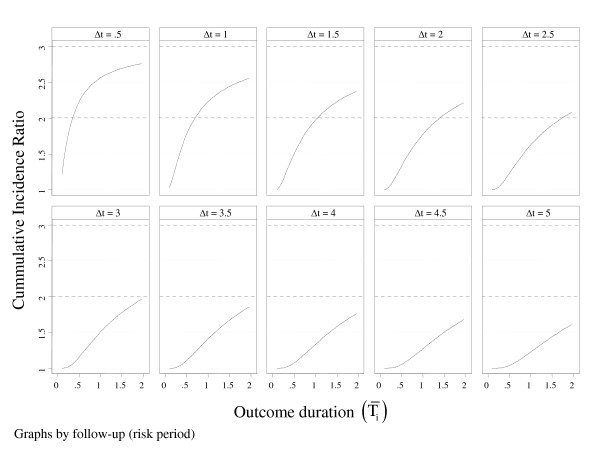
**Projected Cumulative Incidence Ratio (CIR) according to increasing disease durations (${\bar{T}}_{i}$) and increasing risk periods (Δt), given surveys uncovering P1 = 0.5 and P0 = 0.25 (PR = 2.0)**.

The discrepancy between the PR and the CIR may be made more poignantly from yet another angle. Figure 2 extends Figure 1 by showing how the CIR departs from the PR, not only in regards to the outcome duration (${\bar{T}}_{i}$), but also according to the risk period of follow up (Δt) to which the PR is referred to. Placed again within a plausible survey context (*P_1 _*= 0.5 and *P_0 _*= 0.25), Figure 2 portrays 10 Δt-scenarios. As before, on the whole, the detected PRs (= 2.0) imply an immense gamut of CIR estimates. In situations where Δt assumes relatively short cumulative risk periods, the CIRs corresponding to the PR = 2 go to extremes, be it under or overestimating the latter as ${\bar{T}}_{i}$ progressively increases. As the Δt-risk increases, the PR tends to gradually overestimate the CIR. In the 'extreme' Δt = 5 condition (last graph in Figure 2), all surveys detecting a PR = 2.0 would overestimate the CIR whatever the value of ${\bar{T}}_{i}$.

Almost all scenarios depicted in Figure 2 suggest that there are certain combinations wherein the curves cross the lower dotted line demarcating the detected PR. This shows that there is always a prospect of finding a PR estimate that is close to the 'true' CIR in a survey nested into a particular fixed population follow up (cohort), e.g., ${\bar{T}}_{i}$ = 0.4 if Δt = 0.5; or ${\bar{T}}_{i}$ = 1.1 if Δt = 1.5; or ${\bar{T}}_{i}$ = 1.7 if Δt = 2.5; or ${\bar{T}}_{i}$ = 2.0 if Δt = 3.0; or ${\bar{T}}_{i}$ = 2.75 if Δt = 4.0. However, even if one knows something about the outcome's duration (${\bar{T}}_{i}={\bar{T}}_{1}={\bar{T}}_{0}$), it is never possible to specify to which Δt-risk the PR really relates to. Having carried out a survey and estimating the contrast between two prevalences (exposed and unexposed), the researcher will always be in dark as to which time period the risks account for and thus, by extension, the ensuing CIR purportedly emulated by the PR.

**Figure 3:**
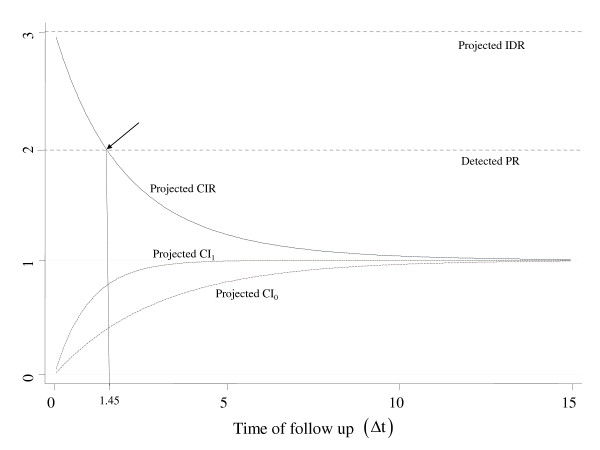
**Projected Cumulative Incidence by exposure group (CI1 and CI0) and ensuing Cumulative Incidence Ratio (CIR), according to increasing risk periods (Δt), given surveys detecting P1 = 0.5 and P0 = 0.25 (PR = 2.0), and **${\bar{T}}_{1}={\bar{T}}_{0}=1$.

At this point one should recall an essential question posed before. In a survey, what would the interpretation of a prevalence contrast be in terms of a CIR (relative risk) on detecting, for instance, a PR = 2.0 as a proxy to a CIR = 2.0 in a scenario akin to scenario 5 of Table 1? One line of thought would be to picture this survey accommodated within another study carried out on a fixed population (cohort) in steady-state and installed in *t_0 _*but already followed for Δt = 1.45 (e.g., year), governed by constant forces of morbidity of ID_0 _= 0.333 and ID_1 _= 1.0 (i.e., IDR = 3). The detected PR = 2.0 would then represent a CIR of 2.0, the estimate that would have been found in *t_1.45 _*if the closed and intact population were effectively followed up for the specified Δt (1.45 year). This situation is signaled with an arrow in Figure 3.

At close scrutiny, though, this quite attractive appraisal is untenable. From the stance of a relative risk (CIR) interpretation, a researcher uncovering a PR = 2.0 cannot know which Δt is at issue. The assumption that Δt = 1.45 is empirically unrecognizable, as is thus the interpretation itself. Counter to a common view, a detected prevalence estimate may not be referred to any risk estimate. The key point is that prevalences have little bearing to the CIR, which comes to show that, beyond any numerical discrepancy, the interpretation of PR in terms of a CIR also implies a conceptual misunderstanding.

Turning to another scenario, what is being measured given the conditions portrayed in Figure 3 when, for instance, a researcher faces a PR = 2.0? Let us focalize, for instance, the particular moment *t_5 _*when Δt = 5. Here, the PR would be attempting to report a situation depicting an underlying force of morbidly of IDR = 3, wherein subjects installed at *t_0 _*were followed through *t_5_*, and upon which the obtained CIR would be 1.225; CI_0 _= 0.811 and CI_1 _= 0.993; and ${\bar{T}}_{1}={\bar{T}}_{0}=1$. This is very different from the PR = 2.0 obtained in the survey at this point in time. Another key issue to emphasize, therefore, is that it is necessary to specify which scenario a PR estimate is referred to, something that is unfeasible in most real life circumstances. Prevalence ratios may well be calculated in a particular cross-sectional study, but within a causal model framework, in general, their interpretation is neither that of risk ratios (CIR) nor of rate ratios (IDR).

The auspicious news, though, is that the directly calculated POR consistently estimates the IDR (equation (10)) if the supporting pillars effectively hold. Given the prevalence used in all 3 figures displayed in this subsection, POR = [*P*_1 _× (1 - *P*_0_)]/[*P*_0 _× (1 - *P*_1_)] = [0.5 × (1 - 0.25)]/[0.25 × (1 - 0.5)] = 3.0, which is consistent with the underlying IDR (top dotted lines). Examining Figure 2 in particular, the equality stands whatever ${\bar{T}}_{i}$ and Δt is involved.

### Choosing a suitable multivariable model

Considering the arguments so far, one has to question the option to unconditionally take the analysis a step further and, without any scrutiny, model the PR. If its meaning in terms of both the CIR and IDR is indefensible in most situations, what would thus be inferable from a non-causal estimate accounting (controlling) for several variables? Apart from very exceptional (and eccentric) circumstances in which a cross-sectional approach were used to study a rare outcome -- when the PR tends to the IDR --, the best answer perhaps should be an uncompromising "not much"! Whichever modeling procedure is used -- whether robust Poisson or Cox, or log-binomial models --, the PR rarely accomplishes its purpose in providing a meaningful estimate within the potential outcomes model framework. On the other hand, if the conditions for causal inference from cross-sectional data are fulfilled and the POR effectively and consistently estimates the IDR, the logistic regression model will provide an unbiased estimate, independently of any "rare disease assumption". It is the 'natural' choice once the potential outcome model is held.

An exception whereby models like the robust Poisson, Cox, or log-binomial may be suitable for modeling data arising from a cross-sectional approach is when one is able to retrospectively reconstitute the entire empirical experience of a fixed population. This may be the case if it is possible to retrieve information by recall, not at all different from a retrospective non concurrent cohort study in which one is recovering the history of a fixed population by way of health service records. This recall would be informing about what happened 'backwards in time' although incident cases taking place at some earlier point would only be counted at the moment of interviewing, irrespectively of when cases occurred between time of inception (*t_0_*) and time of interview (*t_1_*).

One contention is that some subjects -- whether exposed or non-exposed -- would initiate this potential 'closed population' follow up, but would eventually not reach *t_1 _*(for instance, in a sample of women interviewed at birth about an outcome event occurring during pregnancy). However, if this missingness is only conditional on exposure status and not on the outcome (i.e., missing data is random, either completely at random -- MCAR -- or just at random -- MAR [31]), the proportions excluded should be balanced across exposure-outcome combinations, so that the frequency of outcome cases remains the same across exposure groups. Accordingly, the subjects accrued and observed at *t_1 _*do not stand for prevalent and non prevalent cases (by exposure group), but rather provide unbiased information on incidence as in the 'complete' cohort that would have been installed before any withdrawal took place (as abortion would be in the example above).

Thus, if the sample is complete or non differential missingness holds, the design may be characterized as a classical 'fixed population' retrospective cohort, with exposed and non-exposed cohorts installed at *t_0_*; outcomes occurring along Δt and being eventually measured and counted at *t_1_*. This typifies incident cases and thus the causal measure of interest should be the CIR rather than the PR. To emphasize, although the approach is cross-sectional (at *t_1_*), information concerns a cohort moving through time. Also note that, besides an identifiable inception moment -- *t_0 _*--, there is also a clearly identifiable follow-up period -- Δt -- as there should be to effectively obtain a CIR.

### Decision tree for analyzing cross-sectional data

**Figure 4:**
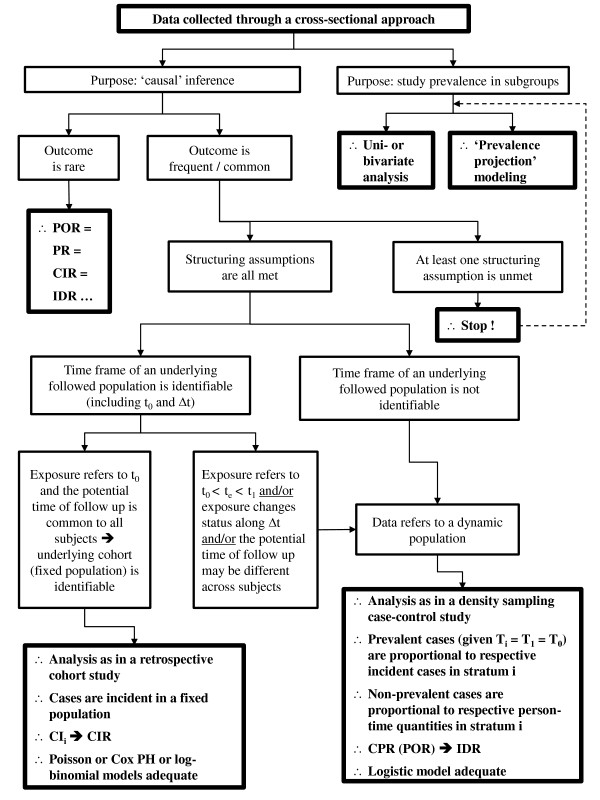
**Decision tree for analyzing cross-sectional data**.

Building on the arguments provided so far, Figure 4 proposes a set of steps to be followed on deciding which measure to use when data is collected through a cross-sectional approach. If the purpose is genuinely to study prevalences in population subgroups, then simple uni or bivariate analysis will suffice. As presented in the section on the purpose of cross-sectional studies, sometimes probability prediction models will also be useful.

Yet, given the aim is to use a cross-sectional approach to assess causality and the outcome is rare, whichever estimator is chosen will be adequate. Alas, rare outcome events are hardly ever studied in surveys. Outcomes are usually common and in this case, a crucial first step is to ask whether the structuring assumptions previously outlined are actually met. If not, there is little one can do and for practical reasons one has to clearly opt for a descriptive perspective at the most (although this is not less important from a public health perspective).

If all conditions are met -- population is in steady state/stationary; there is no selective survival; mean duration is the same in both exposure groups; there is no reverse causality; and temporal directionality from the exposure to the outcome is sustainable --, the researcher has then to figure out if there is enough information available to recognize the time frame of the underlying population, including several time-related references such as *t_0 _*and Δt.

If there is information on this time frame, the next step is to identify which variable is under focus as the exposure of interest in the analysis. If the researcher is fairly confident that the studied exposure is referred to the beginning of the presumed *de jure *reconstituted follow up window and the individual risk periods are definable, then the analysis is analogous to a retrospective cohort study. For instance, if birth weight is the exposure of interest (*vis-à-vis *a childhood development benchmark as outcome such as sitting unaided) and is retrieved from mothers of children one year of age, the researcher may be quite confident that s/he is capturing information regarding an exposure at the inception of the cohort that is being 'reconstituted' -- birth -- and that a recognizable 'closed' follow up period is demarcated -- 1 year. In so being, the measure of interest is the CIR and the robust Poisson, Cox or log-binomial models are perhaps the most suitable ensuing multivariable models.

Another possibility is that the exposure of interest may not be informative of an event occurring at *t_0 _*and started someway along the reconstructed follow up period (Δt). Given that the timing of exposure occurrence *t_e _*lies within Δt (*t_0 _*<*t_e _*<*t_1_*), individual follow up times may no longer be equal and as a consequence some subjects would not be observed for the whole risk period. Since this is equivalent to an unequal person-time apportionment in a prospective study design, the possibility of studying risk (CI*_i_*) is no longer possible. The same applies if the exposure of interest changes status along Δt and/or if the potential time of follow up varies across subjects.

Strictly, these scenarios are akin to that obtained in the vast majority of cross-sectional studies whereby data refers to a dynamic population and the time frame of an underlying followed up population is simply not recoverable. In all these situations, provided the structuring assumptions are tenable, the analysis proceeds as in a density sampling case-control study wherein non-prevalent cases are proportional to respective person-time quantities and ultimately sustain an unbiased estimate of the IDR through the calculated cross-product ratio [30,32]. Hence, as previously mentioned, the logistic model may be the most suitable related multivariable model.

### Putting it all together

In the light of our own contentions, it is worth re-examining the dispute 'between camps' alluded to in the introduction. As mentioned, several arguments against the POR have been raised [13-18]. One is that the POR is difficult to interpret and communicate. According to Pearce [19], Strömberg [12] and our own arguments, the POR should not be difficult to interpret once it is understood that the POR is an acceptable measure to estimate the IDR, which, in turn, also implies understanding what the quantities represented by the prevalent and non-prevalent cases stand for (akin to a density sampling case-control study [20,33]).

Another argument against the POR was that it is very discrepant from PR when outcomes are common. We stand with Pearce [19] in contending that "*the fact that the two methods give different results when the disease is common *[...] *does not tell us which measure is more appropriate to use*". We go beyond this assertion by putting forward that it is in fact a positive aspect that the POR is discrepant from the PR when the outcome is common since, except for very restrictive and most unrealistic circumstances, the latter does not stand for much as a causal parameter, whereas the former may be meaningful (namely, standing for the IDR) given certain conditions -- e.g., stationarity and equal duration of disease. Although, according to Thompson [18], these conditions are hardly met and would thus disfavor the POR, it is by no means clear how the PR would survive this criticism as well, since it is also strongly affected by any violation of these assumptions.

Yet another argument against the POR within the context of common outcomes is that the measure is at times misinterpreted as a cumulative incidence ratio (CIR). In our view, it does not seem fair to blame the measure instead of who misunderstands it. Moreover, we believe that the same risk of misinterpretation holds for the PR.

The remark made by some authors such as Lee [15] that the only usefulness of POR is to mimic other ratio measures should not be taken as a criticism, but conversely, as a positive facet. If anything, it is auspicious that the cross product ratio generated by way of a cross-sectional approach is able to provide a contrast of incidence measures in some conditions, which is unlikely to occur with the PR. By extension, coefficients obtained in a logistic regression should be regarded as multivariate cross-product ratios that, given a causal framework, stand for unbiased estimates of IDRs.

Beyond the points debated so far, Thompson et al. [18] additionally argue that, given the absence of longitudinal data and the inability to make proper causal inferences once cross-sectional data will be used, it is best to use PR because this overtly signals its "*limited inferential value*" and thus warns the reader ensuring "*truth in advertising*". Again, it is our stand that this perspective is essentially ill-advised in regards to etiological inference. First and foremost, conditions for causal inference are either present (assumable) or not. If not, there is not much reason for estimating an effect measure and label it as of 'limited inferential value'. The discussion on the best estimator and related model only makes sense if preceded by a thorough debate about what is actually sought with a cross-sectional study and what the ensuing best measures for inference are. If the assumptions for etiological inference do not hold, there is no reason for modeling data in order to control for confounding. Dealing with confounders has no meaning outside a causal reasoning and is only justified under a counterfactual logic [28,34].

The growing literature on alternative multivariable models to estimate effects arising from cross-sectional data, instead of aiding in the understanding and development of epidemiological research, on the contrary, may have brought more shadows than light on the matter. Not because the intricacies of the proposed models are incorrect, but because the importance of actual epidemiological model building have been largely sidestepped. The conditions and appropriateness for modeling are taken for granted, yet as this paper attempts to remind, considering theoretically based putative relations between the events of interest is crucial to the process. Outcomes and exposures, as well as other elements involved in the causal system -- confounders, effect modifiers, mediators, colliders [30,35] -- not only require assessment on matters of substance and meaning, but also in regards to their temporal relations within the time frame of any given study. Only then are decisions to be taken in favor of any statistical model. And given the recognizably restricted situations where causal modeling is indeed obtainable from cross sectional data, the POR -- or rather, the calculated cross product ratio as an estimate of the IDR -- ought to be the most widely indicated estimator.

## Summary

During the last two decades the choice between Prevalence Ratio (PR) and Prevalence Odds Ratio (POR) to contrast exposure groups in cross-sectional studies has been the subject of debate. In the last 10 years, this debate became more focused on the choice between different regression models for estimating PR. In this paper we used different scenarios to illustrate and sustain our point of view concerning two issues related to the analysis of cross-sectional data. Firstly, when conditions for causal inference in cross-sectional studies do not hold, crude or subgroup prevalences are the quantities to be presented. Secondly, when the assumptions for causal inference are met, two additional aspects need to be considered: (i) cumulative incidence ratio (CIR) is not properly estimated using either PR or POR; (ii) the only measure that provides an unbiased estimate of incidence density ratio (IDR) is the POR. An exception for these two statements is the presence of rare diseases, which usually are not subject of surveys. Based on these facts, we sustain that multivariate modeling should be restricted to scenarios were assumptions for causal inference from cross-sectional studies effectively hold and that for such cases the logistic regression model remains the appropriate choice to capture the incidence contrast between exposure groups.

## Abbreviations

CI: Cumulative incidence; CIR: Cumulative incidence ratio; ID: Incidence density; IDR: Incidence density ratio; OR: Odds ratio; P: Prevalence; POR: Prevalence odds ratio; PR: Prevalence ratio; RR: Risk ratio.

## Competing interests

The authors declare that they have no competing interests.

## Authors' contributions

Both authors (MER and ESFC) made substantial contributions to conception, analysis and interpretation of the data. Following discussions on the outline of the paper both authors made separate contributions to the first drafting of the manuscript and, thereafter, interactively participated in critically revising the content up to the final (submitted) version. MER was responsible for setting up the scenarios and generating the computer programs (routines) for the analysis and entailing displays (tables and figures). The authors read and approved the final manuscript.

## Pre-publication history

The pre-publication history for this paper can be accessed here:

http://www.biomedcentral.com/1471-2288/10/66/prepub
